# Epidermal stem cells are retained *in vivo* throughout skin aging

**DOI:** 10.1111/j.1474-9726.2008.00372.x

**Published:** 2008-04-01

**Authors:** Adam Giangreco, Mei Qin, John E Pintar, Fiona M Watt

**Affiliations:** 1 Cancer Research UK, Cambridge Research Institute Li Ka Shing Centre, Robinson Way, Cambridge CB2 0RE UK; 2 Department of Neuroscience and Cell Biology, University of Medicine and Dentistry of New Jersey Piscataway, NJ 08854 USA

**Keywords:** aging, epidermis, Igf, Igfbp, skin, stem cell

## Abstract

In healthy individuals, skin integrity is maintained by epidermal stem cells which self-renew and generate daughter cells that undergo terminal differentiation. It is currently unknown whether epidermal stem cells influence or are affected by skin aging. We therefore compared young and aged skin stem cell abundance, organization, and proliferation. We discovered that despite age-associated differences in epidermal proliferation, dermal thickness, follicle patterning, and immune cell abundance, epidermal stem cells were maintained at normal levels throughout life. These findings, coupled with observed dermal gene expression changes, suggest that epidermal stem cells themselves are intrinsically aging resistant and that local environmental or systemic factors modulate skin aging.

## Introduction

Skin aging involves increased susceptibility to injury and infection, reduced wound healing, loss of dermal elasticity, poor epidermal barrier maintenance, wrinkling, hair loss, and increased cancer risk (McCullough & Kelly, 2006). Normal skin homeostasis is maintained by epidermal stem cells that reside in protective microenvironments where they self-renew and produce daughter cells that undergo terminal differentiation (Watt & Hogan, 2000; Watt *et al*., 2006). Stem cells signal to each other within the skin via both cell–cell contact and diffusible factors (Jamora *et al*., 2003; Hobbs *et al*., 2004; Kobielak & Fuchs, 2006). Epidermal stem cells are considered the likely origin of cancers as their high-proliferative capacity and longevity allow them to accumulate oncogenic transforming mutations (Owens & Watt, 2003).

Based on observations in haematopoietic and other tissues, it has been suggested that stem cells directly regulate tissue aging (Rossi *et al*., 2005; Rando, 2006). Despite clear evidence that aged skin accumulates senescence markers (such as p16/Ink4a) (Ressler *et al*., 2006), it remains controversial whether epidermal stem cells intrinsically age. *In vitro* culture studies suggested that aged mouse epidermal keratinocytes function equivalently to those isolated from young animals (Stern & Bickenbach, 2007). In contrast, skin cells isolated from elderly humans and cultured using similar conditions contained fewer stem-like cells (Barrandon & Green, 1987). Recently, genetically engineered telomerase-deficient mice were generated which exhibited characteristics of premature skin aging (Flores *et al*., 2005). Telomerase-deficient epidermal stem cells exhibit poor *in vitro* clonogenicity and are unresponsive to mitogen stimulation *in vivo*. However, loss of telomerase activity is not confined to the epidermis in these mice, and it remains unclear whether these changes are caused by stem-cell-intrinsic telomerase deficiency.

In addition to chronologic aging, studies have investigated the effects of UV irradiation on skin aging and cancer susceptibility (known as photo-aging) (Yaar & Gilchrest, 2001; McCullough & Kelly, 2006). Because of the UV resistance of hair-bearing mammals, most of these studies have involved the use of hairless mouse mutants (Hr/Hr and others) (Kligman, 1996). Photo-aging models have demonstrated that both epidermal stem cells and their progeny can accumulate DNA damage upon UV irradiation, although no studies have addressed whether this damage alters intrinsic stem cell functionality (Nijhof *et al*., 2007).

The purpose of the current study was to characterize changes in murine skin homeostasis and stem cell abundance during normal aging. We determined that epidermal stem cells are retained throughout life despite significant age-associated changes in dermal thickness, epidermal proliferation, and peripheral immune cell abundance. These findings suggest that local environmental rather than stem-cell-intrinsic factors influence skin aging.

## Results

### Altered dermal and epidermal morphology is associated with skin aging

**Fig. 1:**
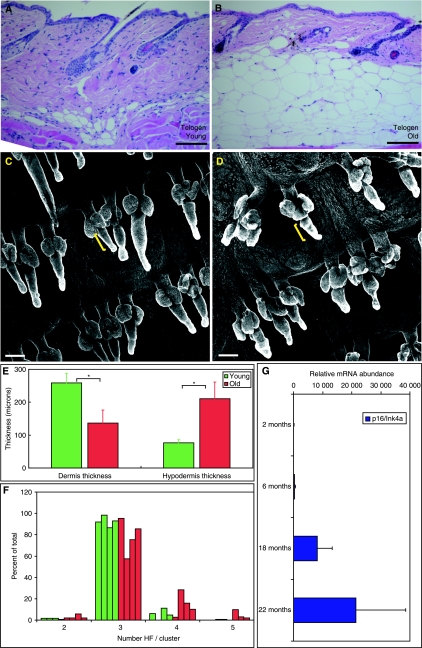
Age-associated changes in murine skin. (A, B) Haematoxylin-and-eosin-stained sections of young (A) and old (B) telogen murine dorsal skin showing epidermis, dermis, hypodermis, and underlying muscle. Abnormal follicular architecture, dermal thinning, and hypodermal thickening are present in aged skin. (C, D) Keratin 14-stained skin whole-mount images from young (C) and old (D) mice [bracket indicates hair follicle (HF) bulge]. (E) Average dermis (measured from epidermis to hypodermis) and hypodermis (measured from dermis to underlying muscle) thickness in young (green) and old (red) mice. (F) Average number of HFs per cluster in young (green) and old (red) tail epidermis. (G) Quantitative polymerase chain reaction analysis of p16/Ink4a/Arf gene expression in skin of mice of increasing age. Scale bars = 100 µm (A, B, D, E). (*n* = 4 mice/age; *P < 0.05).

In order to determine whether murine skin exhibited any overt changes with increased age, we obtained young (2–6 months) and old (22–26 months) adult C57/Bl6 mice from the National Institute of Health (NIH) and an in-house colony. Telogen-phase mouse dorsal skin from 24-month-old to 26-month-old mice presented significantly decreased dermal cellularity and thickness, while the subcutaneous adipose layer (hypodermis) was increased relative to young telogen-phase dorsal skin (Fig. 1A,B,E). The total dermal plus hypodermal thickness was similar in young and old skin, raising the possibility that dermal to hypodermal conversion had occurred (Fig. 1E).

Age-associated epidermal changes were detected following tail whole-mount keratin 14 antibody staining. These included hair follicle (HF) swelling and loss of normal sebaceous gland architecture (Fig. 1C,D). Old mice also exhibited a variable loss of normal HF triplet patterning relative to young controls (Fig. 1F). Despite these morphological changes, the stem-cell-containing ‘bulge’ was retained within most aged HFs (yellow brackets, Fig. 1C,D). Skin p16/Ink4a/Arf expression increased during aging, confirming previous observations that this transcript serves as a biomarker of aged skin (Fig. 1G; Ressler *et al*., 2006).

### Epidermal stem cell abundance is unaffected by skin aging

**Fig. 2:**
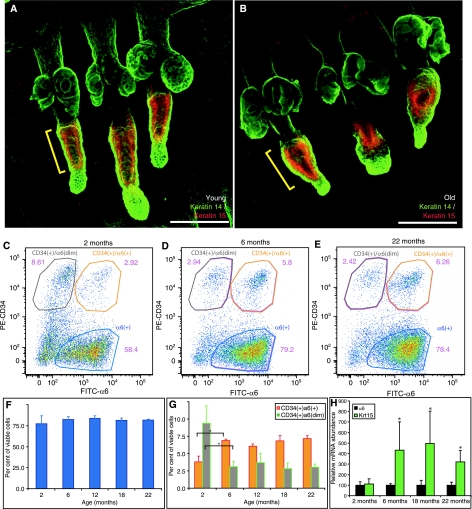
Epidermal stem cells are maintained during skin aging. (A, B) Representative epidermal whole mounts from young and old mice stained for keratin 14 (green) plus the stem cell marker keratin 15 (red) to identify hair follicle bulge stem cells. (C–E) Representative flow cytometry plots from 2-month-old (C), 6-month-old (D), and 22-month-old (E) murine epidermal skin preparations stained for CD34-PE and α6 integrin–FITC. Three distinct epidermal populations are shown: α6(+) basal cells (blue gate), α6(+)/CD34(+) stem cells (orange gate), and α6(dim)/CD34(+) cells (grey gate). (F) Quantification of the per cent of viable cells represented within the α6(+) gated population from epidermal preparations of *n* = 3 mice/age at each of 2 months, 6 months, 12 months, 18 months, or 22 months. (G) Quantification of the per cent of viable cells represented as α6(+)/CD34(+) stem cell (orange bars) or α6(dim)/CD34(+) stem cell (grey bars) populations. No significant differences in stem cell abundance were observed between 6 months and 22 months age. (H) Quantitative polymerase chain reaction analysis of whole-skin RNA for α6 (black bars) and keratin 15 (green bars) levels in 2-month-old, 6-month-old, 18-month-old, and 22-month-old mice. Scale bars = 100 µm (A, B). Asterisk (G, H) indicates significant difference versus 2-month sample; *P* < 0.05.

The observation that the ‘bulge’ region was retained prompted us to examine whether epidermal stem cell abundance was altered within aged skin. To determine this, we performed whole-mount immunostaining using antibodies directed against the bulge stem cell protein keratin 15 (Liu *et al*., 2003). Despite age-associated follicular morphological changes (first described in Fig. 1), we determined that keratin 15(+) bulge stem cells were retained in follicles of both young and old mice (Fig. 2A,B).

We also quantified epidermal stem cell abundance by flow cytometry of cells labelled with antibodies to CD34 and integrin α6 (Trempus *et al*., 2003). We determined that although CD34(+)/α6(+) stem cells increased between 2 months and 6 months of age, epidermal stem cell abundance was unchanged in 6-month-old, 12-month-old, 18-month-old, and 22-month-old skin samples (representative plots shown in Fig. 2C–E; quantified in Fig. 2F,G). Two- to six-month age-associated differences in CD34(+)/α6(dim) and CD34(+)/α6(+) epidermal stem cell abundance likely reflect the previously described transition between suprabasal and basal follicular stem cell populations (Blanpain *et al*., 2004). Keratin 15, but not α6 integrin, mRNA expression increased significantly between 2 months and 6 months of age, and was subsequently maintained throughout aging, in agreement with the immunostaining results (Fig. 2H). Taken together, these findings suggested that epidermal stem cell abundance was not affected by skin aging.

### Epidermal proliferation decreases in an age-dependent, stem-cell-independent manner

**Fig. 3:**
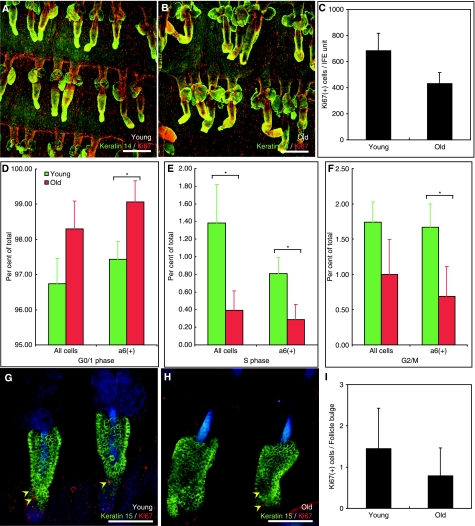
Interfollicular epidermal proliferation declines with aging. (A, B) Representative young (A) and old (B) epidermal whole mounts stained for keratin 14 (green) plus Ki67 (red). (C) Quantification of Ki67(+) nuclei per interfollicular epidermis unit (defined in Silva-Vargas *et al*., 2005) in young and old mice. (D–F) Flow cytometric analysis of total (left side graphs, C–E) or α6(+) keratinocyte (right side graphs, C–E) cell cycle status in young (green) versus old (red) skin preparations. (G, H) Keratin 15 (green) plus Ki67 (red) immunostaining in young and old mice to determine bulge stem-cell-specific proliferation. (I) Quantification of Ki67(+) cells per bulge in young and old mice. Scale bars (A, B) = 100 µm. (*n* = 4 mice/age; *P < 0.05).

To determine whether epidermal proliferation was altered in aged skin, we performed whole-mount immunostaining for Ki67 and keratin 14 (Fig. 3A,B). Proliferation of each unit of interfollicular epidermis (IFE) (as defined by Silva-Vargas *et al*., 2005) appeared to decrease in all aged skin samples, although this was not statistically significant (*P* < 0.09; Fig. 3C). Tail whole-mount labelling for Ki67 revealed that there were no differences in K15 positive, bulge-associated stem cell proliferation between young and old samples (yellow arrowheads, Fig. 3G–I). Nevertheless, flow cytometry revealed an increase in G1 phase cells concomitant with reductions in S and G2/M-phase cells in aged telogen-phase skin [total or α6(+) keratinocytes; Fig. 3D–F]. This is consistent with a modest decrease in proliferation with age.

### Altered peripheral immunity in aged skin

**Fig. 4:**
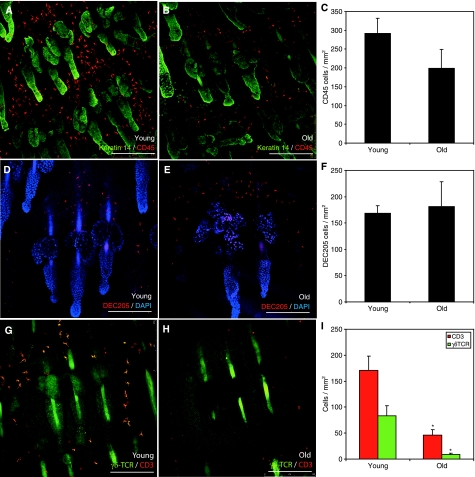
Aging results in altered skin leukocyte abundance. (A, B) Representative whole mounts from young (A) and old (B) tail epidermis stained for the pan-haematopoietic marker CD45 (red) and the keratinocyte-specific marker keratin 14 (green). (C) Quantification of CD45(+) cells present per square millimetre of tissue. (D, E) Young (D) and old (E) epidermal whole mounts stained to reveal haematopoietic Langerhans cells (DEC205, red). (F) Quantification of DEC205(+) cells per square millimetre of epidermis. (G, H) Whole-mount immunostaining for T cell populations using antibodies to the pan-T cell marker CD3 (red) and γδ-T cell receptor (γδ-TCR; green) to identify dendritic epidermal T cells (DETCs, orange dual stain). (I) Quantification of CD3 (red) and γδTCR/CD3 dual (green) positive cells per square millimetre of epidermis. All images and quantification represent at least *n* = 3 individuals/age. Scale bars = 200 µm (A, B); 100 µm (C–F).

In addition to altered morphology, skin aging is frequently associated with reduced peripheral immunity and increased infection (McCullough & Kelly, 2006). To study whether aging influences murine skin immunity, epidermal whole mounts were stained for the pan-haematopoietic cell antigen CD45. There appeared to be fewer CD45 reactive cells within the IFE of old mice but this was not statistically significant (Fig. 4A–C). In young but not old mice, most of these CD45 positive cells exhibited a dendritic morphology. CD45 positive cells present in old skin were localized to the follicular–interfollicular junction (infundibulum; Fig. 4A,B).

To further characterize the haematopoietic cells present in young and aged skin, we performed immunostaining to identify Langerhans cells (DEC205 reactive), T cells (CD3 reactive), and atypical dendritic epidermal T cells (DETCs, γδTCR reactive). There were no significant differences in Langerhans cell abundance or localization between young and aged skin (Fig. 4D–F). In contrast, there was a dramatic and statistically significant loss of both epidermal T cells (CD3) and DETCs (CD3+ γδTCR) associated with skin aging (Fig. 4G–I). This near-complete loss of DETCs despite retention of rare CD3(+), αβ T cells suggests that aged murine skin may have impaired innate immunity, and therefore, may be more susceptible to infection following injury.

### Skin aging is associated with reduced Igfbp3 expression

**Fig. 5:**
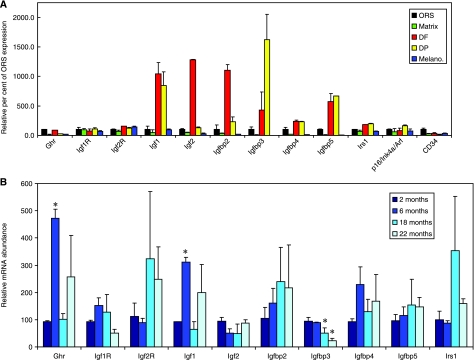
Skin aging results in changes in Igfbp3 transcript abundance. (A) Bioinformatic analysis of relative Igf/Igfbp signalling pathway gene abundance in matrix (green), outer root sheath (ORS, black), dermal fibroblast (DF, red), dermal papilla (DP, yellow), or melanocyte (blue) cell populations relative to ORS/basal keratinocytes. Most Igf pathway members were enriched within dermal compartments (DF and DP). (B) Quantitative polymerase chain reaction of whole-skin cDNA to determine Igf/Igfbp expression in 2-month-old, 6-month-old, 18-month-old, and 22-month-old mice. (*n* = 3 mice/age; *P < 0.05).

Based on our observation that skin aging did not correlate with a reduction in the number of epidermal stem cells, we hypothesized that aging might instead be regulated by environmental components. The Igf signalling pathway has previously been identified as a key mediator of both epidermal proliferation (Edmondson *et al*., 2003) and skin peripheral immunity (Sharp *et al*., 2005a). In addition, Igf/Igfbp signalling is a well-established component of many aging models, where it influences tissue growth, glycolysis, adiposity, and cellular stress resistance (Kenyon, 2001; Holzenberger *et al*., 2003; Tatar *et al*., 2003). Therefore, using existing but previously uncharacterized microarray data (Rendl *et al*., 2005), we compared Igf/Igfbp family member expression within skin dermal fibroblasts (DFs) and dermal papilla (DP) relative to melanocyte, matrix, and outer root sheath (ORS) cells. Most Igf pathway members were strongly (200–1500%) up-regulated in DF/DP cells compared to other cell types, suggesting that skin Igf signalling is primarily dermis derived (Fig. 5A).

To address whether aging resulted in altered Igf pathway expression, we generated cDNA from whole skin of 2-month, 6-month, 18-month and 22-month aged mice for quantitative polymerase chain reaction (QPCR) analysis. Glyceraldehyde-3-phosphate dehydrogenase (GAPDH) and integrin α6-specific TAQman probes were used to normalize input RNA. Ghr and Igf1 abundance significantly increased between 2 months and 6 months, although neither transcript exhibited age-associated expression trends (Fig. 5B). Only Igfbp3 exhibited significantly reduced transcript abundance with increasing chronologic age (Fig. 5B). This fivefold decrease in Igfbp3 from 2 months to 22 months of age is in agreement with previous reports documenting Igfbp3 transcript abundance in aged human skin (Lener *et al*., 2006).

**Fig. 6:**
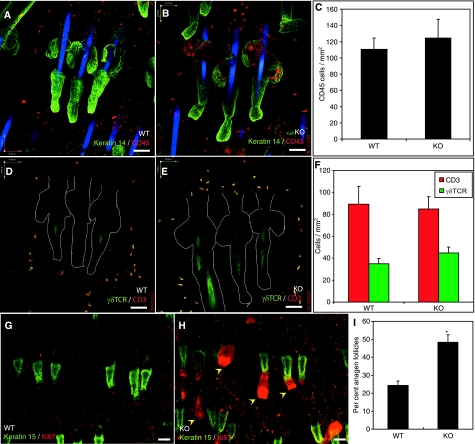
Igfbp3 does not directly influence epidermal immune cell abundance but results in increased anagen follicles. (A, B) Representative whole mounts from age- and sex-matched wild-type (WT) (A) and Igfbp3KO (B) tail epidermis immunolabelled for keratin 14 (green) and CD45 (red). (C) Quantification of CD45 cell abundance in WT and knock-out (KO) epidermis per square millimetre. (D, E) WT (D) and KO (E) whole mounts stained for γδTCR (green) and CD3 (red) identify CD3(+) T cells and dendritic epidermal T cells (orange). (F) Quantification of CD3(+) and γδTCR(+) cell abundance in WT and KO skin. (G, H) WT (G) and KO (H) tail whole mounts stained for keratin 15 (green) and Ki67 (red) to identify bulge stem cells and mitotic keratinocytes, respectively. (I) Quantification of percentage of total growing (anagen) follicles present in WT and KO tail epidermal whole mounts (e.g. see arrowheads, H). Scale bars = 100 µm (A, B, D, E, G, H). (*n* = 4 mice/genotype; *P < 0.05; I).

In order to determine whether altered Igfbp3 expression directly influenced epidermal proliferation or peripheral immunity, we compared these properties in tail epidermal whole mounts from wild-type (WT) and Igfbp3 knock-out (KO) mice (Ning *et al*., 2006). Total haematopoietic cell (CD45; Fig. 6A–C), and T and DETC cell abundance (CD3 and γδTCR; Fig. 6D–F) were unchanged in Igfbp3KO compared to WT mouse skin. There were also no differences in stem cell abundance, interfollicular proliferation, and follicle patterning (Fig. 6G,H; data not shown). However, the proportion of anagen-phase follicles was significantly increased in Igfbp3KO mice compared with WT controls (Fig. 6I; arrowheads Fig. 6H). These results indicate that although peripheral immunity and interfollicular proliferation decline in conjunction with reduced Igfbp3 expression during aging, these events are not causally linked.

## Discussion

In the present study, we have assessed age-associated changes in murine skin. Skin aging resulted in significant dermal thinning, increased hypodermal thickness, altered epidermal architecture and morphology, reduced interfollicular epidermal proliferation, and loss of peripheral immune cell abundance. We were unable to directly attribute any of these effects to loss of epidermal stem cells or altered stem cell proliferation. These findings suggest that local dermal or systemic environmental, rather than intrinsic stem cell alterations, may be the dominant factors that regulate normal skin aging.

One of the principal findings of the current study is that aged epidermis contains significantly fewer T cells and almost no detectable γδTCR(+) DETCs. These cells serve as a first line of defence against environmental damage while maintaining important immunoregulatory and epithelial repair properties (Sharp *et al*., 2005b). This suggests that our currently described reduction in proliferation during aging may instead be directly attributable to loss of DETC-mediated regulation. Interestingly, loss of DETCs has also been associated with increased susceptibility to chemically mediated epidermal carcinogenesis (Girardi *et al*., 2001), suggesting that age-associated DETC loss may additionally increase cancer risk in elderly mice.

The current study shows that aging results in reduced dermal Igfbp3 gene expression. Although loss of Igfbp3 did not directly contribute to any observed age-associated phenotypes, Igfbp3 KO mice did exhibit an increased proportion of growing HFs when compared to WT controls. These data suggest that dermis-derived Igf/Igfbp signalling can regulate epidermal growth and differentiation, most likely by modulating bioavailable Igf (Edmondson *et al*., 2003). In support of this, Igf receptor 1 KO mice develop a thin, disrupted epidermis with reduced HF abundance (Liu *et al*., 1993). Cultured epidermis from Igf1R KO mice exhibits accelerated differentiation, decreased proliferation, and elevated apoptosis (Sadagurski *et al*., 2006). Transgenic involucrin-promoter-driven Igfbp3 over-expressing mice exhibit skin hypoproliferation and HF shortening (Edmondson *et al*., 2005; Weger & Schlake, 2005). We propose that dermis-derived Igfbps sequester Igf proteins and regulate their epidermal bioavailability, thereby regulating epidermal proliferation and differentiation.

In summary, our results demonstrate that epidermal stem cells are retained throughout skin aging, and are consistent with a recent report that murine skin stem cells are intrinsically aging resistant (Stern & Bickenbach, 2007). Our findings also suggest that extrinsic, dermis-derived factors regulate skin aging. These results highlight the need to consider both extrinsic and epidermal cell-intrinsic causes of age-dependent skin alterations.

## Experimental procedures

### Mice

Wild-type, specific pathogen-free C57/Bl6 male mice were obtained directly from pre-aged NIH stocks and were 2 months, 6 months, 12 months, 18 months or 22 months old at the time of delivery. The mice were allowed to acclimatize for 72 h, then sacrificed for experiments presented in this paper. Alternatively, specific pathogen-free C57/Bl6 mice were obtained from in-house stocks and were aged 2–4 months (termed ‘young’) or 24–26 months (‘old’) at the time of sacrifice. Igfbp3KO and corresponding WT controls were maintained in-house at the University of Medicine and Dentistry of New Jersey. Age- and sex-matched, identical strain controls were used for these experiments. All mice were provided access to food and water *ad libitum* throughout their lifetime, and maintained on a 12-h light/dark cycle. The mice were sacrificed by CO_2_ asphyxiation, and samples were immediately fixed, frozen, or processed for further analysis. No experimental procedures were performed on any mice prior to sacrifice.

### Immunohistochemistry and imaging

Tissue was processed for tail epidermal whole-mount immunohistochemistry as previously described (Braun *et al*., 2003). Antibodies used were sourced and used at the following dilutions: rabbit-antikeratin 14 (1 : 1000, Covance, Princeton, NJ, USA), rabbit-anti-Ki67 (1 : 300, Novocastra, Newcastle, UK), mouse-antikeratin 14 (1 : 1000, clone LL002; in-house), rat-anti-Cd45 (1 : 100, BD Pharmingen, Franklin Lakes, NJ, USA), rat-anti-Dec205 (1 : 5; provided by Caetano Reis E Sousa), Armenian hamster-antiγδ T-cell receptor (1 : 50; BD Pharmingen), rat-anti-CD3 (1 : 100; BD Pharmingen), rat-anti-CD34 (biotinylated; 1 : 100; BD Pharmingen), rat-antiα6 integrin (FITC; 1 : 200; BD Pharmingen), and mouse-antikeratin 15 (1 : 100; clone LHK15; in-house). Five-micron haematoxylin and eosin stained tissue sections were obtained after fixation in formal saline, paraffin embedding, and sectioning using an autostainer (TissueTek, Sakura Finetek, Torrance, CA, USA). Images were captured using a Nikon90i (London, UK) upright microscope (H&E sections), Zeiss (Welwyn Garden City, UK) Axiophot laser scanning confocal microscope, or Leica (Milton Keynes, UK) Tandem laser scanning confocal microscope (tail whole-mount images). Dermal thickness measurements were made using Nikon NIS Elements AR software. Brightness and contrast were optimized solely for visualization purposes using Adobe Photoshop.

### Flow cytometry

A single cell suspension of epidermal cells was prepared as previously described (Silva-Vargas *et al*., 2005), and cells were selected for analysis on the basis of moderate forward/side scatter properties and 7AAD dye exclusion. Stem cell analyses and staining to assess cellular proliferation were performed as previously described (Trempus *et al*., 2003).

### Bioinformatics

All data for bioinformatics analysis were obtained by examining the publicly available GEO Profile data set GDS1323 corresponding to 4-day-old mouse skin cell fractions isolated by Rendl *et al*. (2005). GEO Profile data set probes corresponding to specific genes were as follows: Ghr (1451501), Igf1R (1452108), Igf2 (1448152), Igf2R (1424112), Igf1 (1437401), Igfbp2 (1454159), Igfbp3 (1423062), Igfbp4 (1423757), Igfbp5 (1452114), Irs1 (1423104), p16/Ink4a/Arf (1450140), and CD34 (1416072). The average MAS5-calculated signal intensity of replicate samples of matrix, ORS, DF, DP, and melanocyte cells was determined for each gene/probe, and presented as a relative percent value versus the average ORS probe signal intensity.

### qPCR

Total RNA was isolated from whole skin using standard phenol:chloroform extraction followed by mRNA purification (polyAtract; Promega, Madison, WI, USA). Quantitative polymerase chain reaction was performed under standard conditions using an ABI7900 real-time PCR machine. All samples were run as triplicates with three samples per age. Quantitation was based on ΔΔCt calculations, and all samples were compared against GAPDH as a loading control and normalized against alpha-6 integrin expression levels to account for variable epidermal:dermal isolation efficiency. TAQman pre-designed probes were purchased from Applied Biosystems (Foster City, CA, USA).

### Statistics

Error bars shown represent the standard error of the mean of between *n* = 3–6 replicates per age and genotype (Igfbp3WT or KO). Error bars in Fig. 5 represent standard deviation as determined by GEO profile MAS5 signal intensity. Over 100 HF clusters were counted per individual mouse (*n* = 4) for HF cluster analysis. The Student's *t*-test was used to compare all samples, and statistical significance was accepted at *P* < 0.05.
